# Postoperative Abulia Following Resection of Bilateral Anterior Cingulate Gyrus Glioblastoma: A Case Report and Lessons Learned

**DOI:** 10.7759/cureus.87547

**Published:** 2025-07-08

**Authors:** Narushi Sugii, Goichiro Tamura, Eiichi Ishikawa

**Affiliations:** 1 Department of Neurosurgery, Institute of Medicine, University of Tsukuba, Tsukuba, JPN; 2 Department of Neurosurgery, Mito Saiseikai General Hospital, Mito, JPN

**Keywords:** abulia, akinetic mutism, anterior cingulate cortex, glioblastoma, salience network

## Abstract

Glioblastoma is a highly malignant brain tumor with a dismal prognosis that requires multidisciplinary treatment, emphasizing 'maximal safe resection' during surgery. Neurosurgeons often rely on empirical knowledge, which suggests that removing only contrast-enhancing lesions should not cause new neurological deficits. However, we encountered a case in which this assumption did not hold true. A 60-year-old, right-handed man presented with declining frontal lobe function and a large mass involving the bilateral anterior cingulate gyrus (ACG) and left superior frontal gyrus (SFG). After initial removal of the left SFG tumor, the patient showed no new deficits. The pathological diagnosis confirmed glioblastoma, IDH-wildtype. A second surgery was performed to resect the remaining contrast-enhancing lesions involving the bilateral dorsal anterior cingulate cortex (dACC), via the initial surgical corridor. Postoperatively, the patient developed persistent abulia. This case highlights a critical exception to prevailing surgical dogma. Although the resection targeted only contrast-enhancing tissue, the involvement of the bilateral dACC, a key node of the salience network, likely led to severe cognitive and motivational dysfunction. Greater attention to functional neuroanatomy may be necessary to optimize outcomes in complex glioblastoma cases, even within contrast-enhancing tumor regions.

## Introduction

Glioblastoma is one of the most devastating brain tumors, with a median overall survival of <20 months [1,2]. The treatment requires multidisciplinary therapy combining surgery, radiation, and chemotherapy; particularly, maximizing the extent of resection while preserving function, termed 'maximal safe resection', is crucial [2,3].

The dorsal anterior cingulate cortex (dACC) is involved in cognitive, emotional, and social behavioral regulation [4]. Damage to the dACC may lead to symptoms such as abulia or akinetic mutism, characterized by decreased responsiveness to surroundings, resulting in mutism and immobility [5,6]. Apathy, abulia, and akinetic mutism are all considered to be part of a spectrum of disorders indicating diminished motivation; the difference lies in severity, with apathy being the mildest and akinetic mutism the most severe form [7].

Although no definitive scientific papers support this, a neurosurgical rule of thumb suggests that removing only contrast-enhancing lesions in glioblastoma does not cause major neurological deficits. However, we encountered a case of glioblastoma involving the bilateral anterior cingulate gyrus (ACG), left medial frontal cortex, and anterior body of the corpus callosum, in which abulia developed after excision of the contrast-enhancing lesion. Herein, we report this case along with a literature review.

We obtained informed consent for publication and anonymized the patient's clinical information in compliance with the Declaration of Helsinki and Japan’s Personal Information Protection Act.

## Case presentation

**Figure 1:**
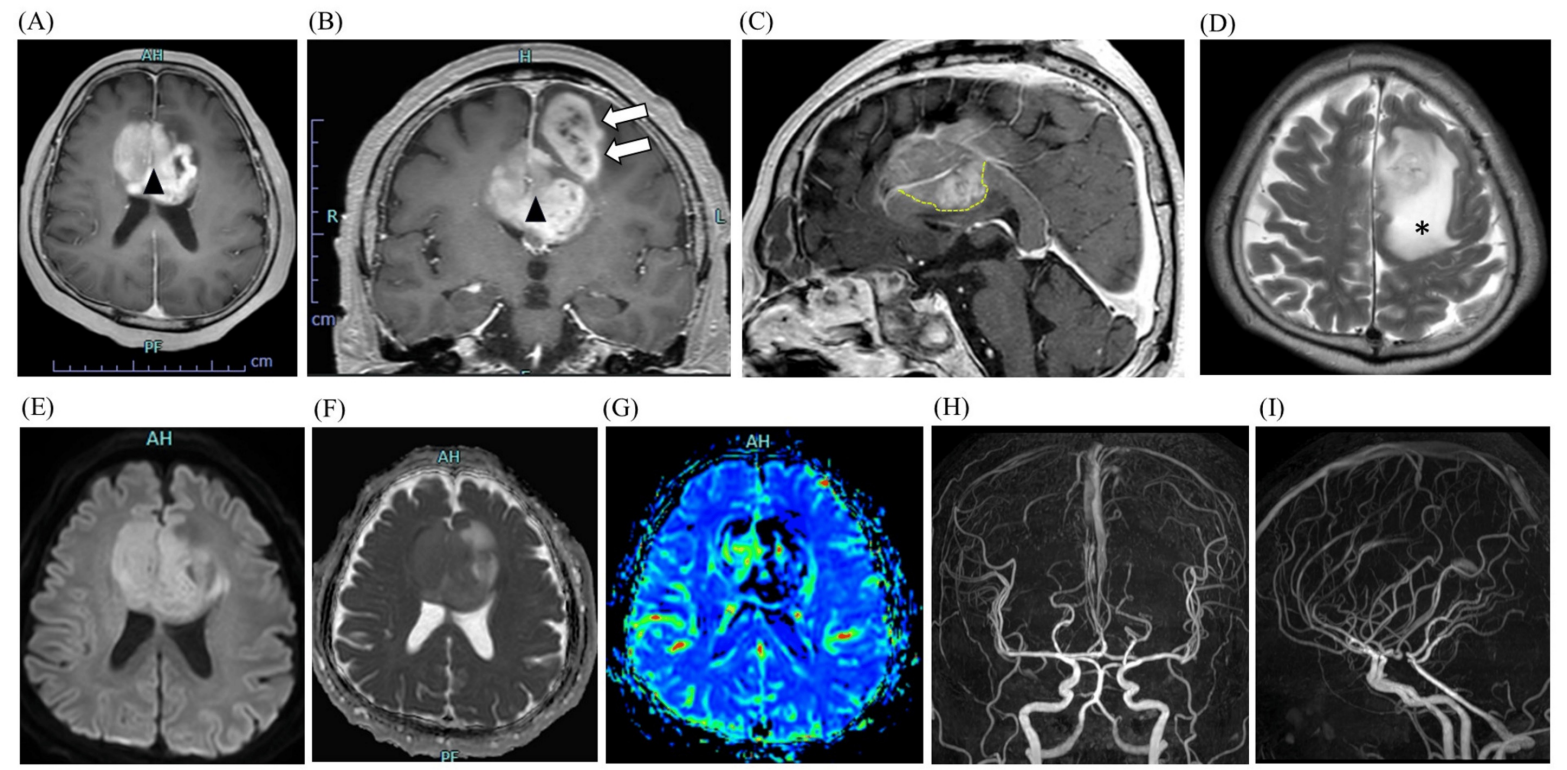
Preoperative MRI findings. Initial brain MRI demonstrates well-enhanced mass lesions (maximum diameter: 65 mm) involving the bilateral anterior cingulate gyri (arrowheads) and the left superior frontal gyrus (blank arrows) on post-contrast T1-weighted axial (A), coronal (B), and sagittal (C) images. Notably, the tumor extends into the anterior body of the corpus callosum (C, dotted line), with surrounding edema visible on the T2-weighted image (D, asterisk). Additional sequences reveal mild diffusion restriction on the diffusion-weighted image (E) and the apparent diffusion coefficient map (F), along with mildly elevated cerebral blood volume as assessed by DSC-MRI (G). TOF-MRA frontal (H) and lateral (I) views show no apparent abnormalities in the main arteries. DSC: Dynamic susceptibility contrast; MRA: Magnetic resonance angiography; TOF: Time-of-flight.

A 60-year-old, right-handed man presented to our hospital complaining of a decline in frontal lobe function, reduced speech frequency, and diminished spontaneity. MRI revealed large (maximum diameter: 65 mm) mass lesions primarily involving the bilateral ACG and left SFG. The lesion exhibited ring-shaped contrast enhancement with surrounding edema extending to the anterior corpus callosum (Figures 1A-1D). Given the mild diffusion restriction and slightly elevated cerebral blood volume without apparent feeding arteries (Figures 1E-1I), the differential diagnosis included malignant lymphoma or high-grade glioma. We initially resected only the left SFG lesion for two reasons: to alleviate intracranial pressure by reducing tumor mass effect, and to secure a surgical corridor for potential second-stage resection if histopathology confirmed high-grade glioma.

**Figure 2:**
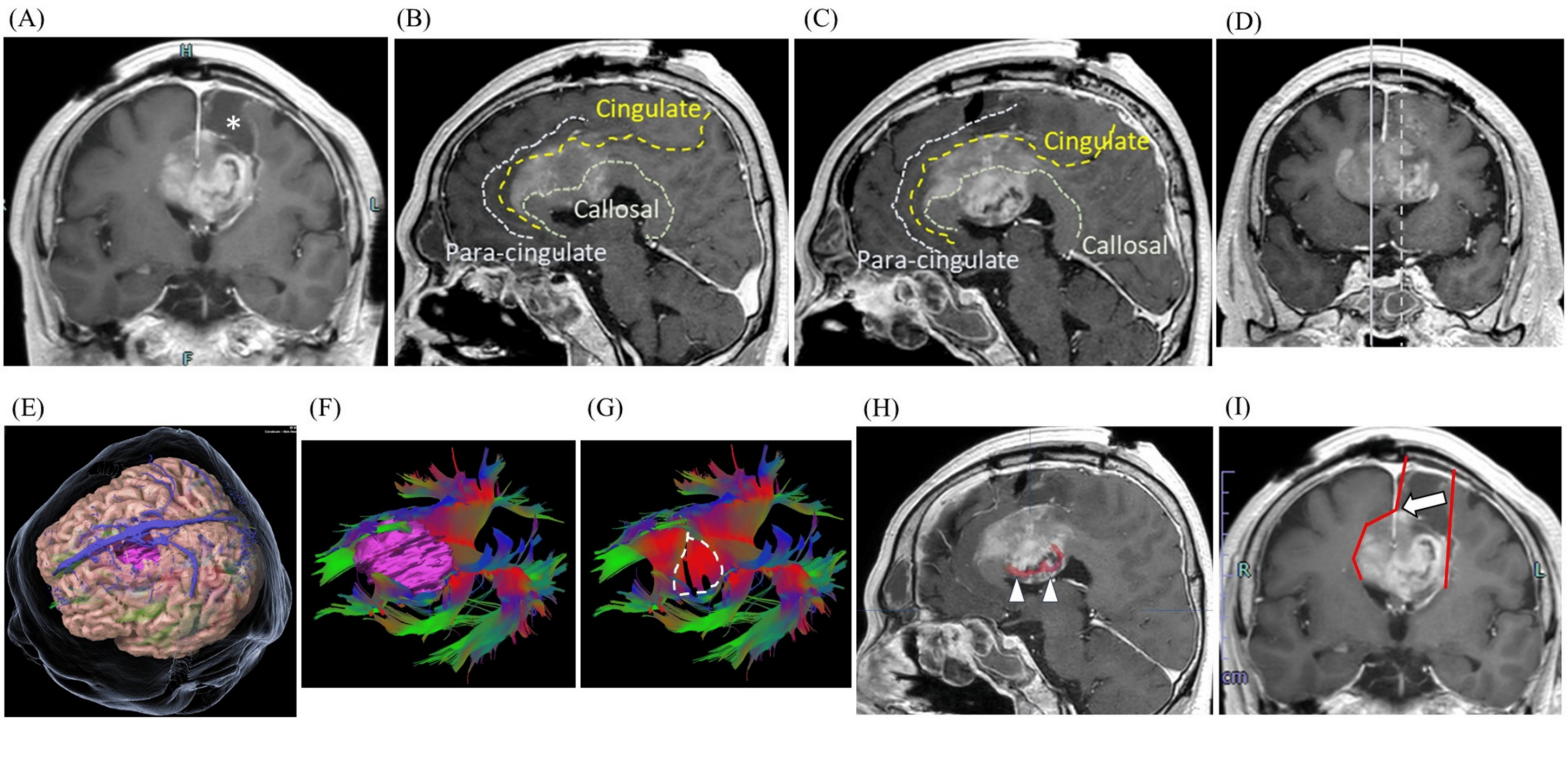
Postoperative MRI findings (initial surgery). Postoperative brain MRI after the initial surgery demonstrates the surgical cavity in the left superior frontal gyrus (asterisk) and residual tumor on the post-contrast T1-weighted coronal image (A). Post-contrast T1-weighted sagittal images (B) and (C) show tumor involvement, with right (solid line) and left (dotted line) midline demarcation indicated in image (D). The paracingulate, cingulate, and callosal sulci are marked by dotted lines in (B) and (C). A three-dimensional image of the brain and translucent skin with the enhancing lesion (pink object) is shown in (E); tractography viewed from the same angle illustrates white matter fibers passing through the corpus callosum (CC) with (F) or without (G) the contrast-enhancing lesion (pink object). Commissural fibers passing through the CC within the contrast-enhanced lesion are also depicted (dotted circle in G; arrowheads in H). Intraoperative approach: the falx cerebri incision (blank arrow) provided access to the contralateral lesion (I). CC: Corpus callosum; WI: Weighted image.

The left SFG tumor was successfully resected as planned (Figure 2A). Postoperatively, the patient exhibited no new neurological deficits and maintained the ability to engage in simple conversations and perform activities of daily living under ward supervision. Histopathological analysis confirmed glioblastoma, IDH-wildtype, CNS WHO grade 4, prompting a decision to proceed with maximal safe resection of the remaining contrast-enhancing lesions under general anesthesia with fluorescence guidance using 5-aminolevulinic acid. Despite bilateral extension of the lesion into the ACG and dACC (Figures 2B-2D), we considered that resection limited to contrast-enhancing areas could be performed safely without causing new deficits. Using the surgical cavity from the initial surgery as a corridor, we divided the falx cerebri to access the contralateral (right) lesions (Figure 2I).

**Figure 3:**
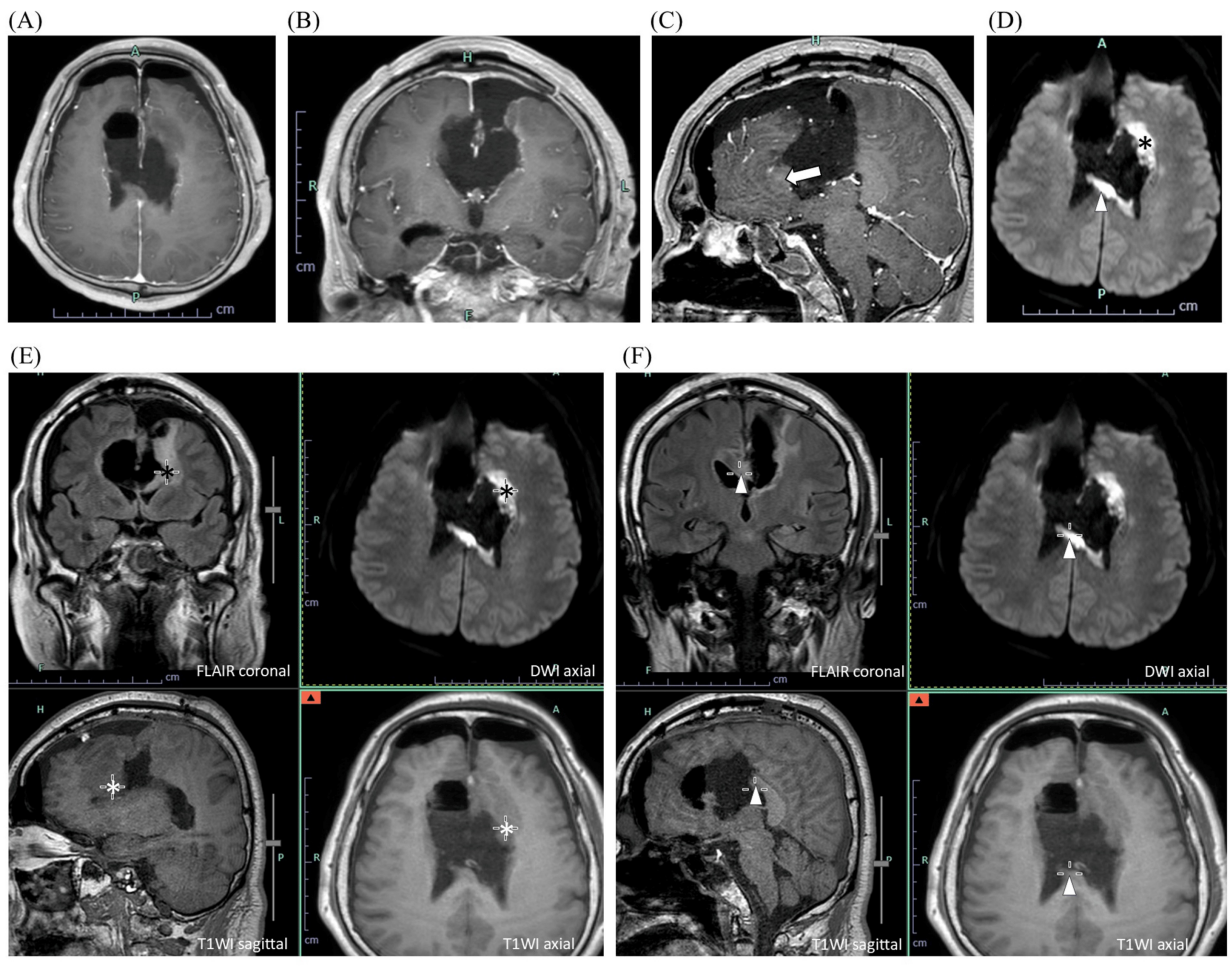
Postoperative MRI findings (second surgery). Postoperative brain MRI after the second surgery demonstrates near-total resection of the enhancing lesions on post-contrast T1-weighted images (T1WIs) in axial (A), coronal (B), and sagittal (C) sequences. The genu of the corpus callosum appears structurally intact (blank arrow); however, DWI reveals cerebral infarction involving the genu (asterisks) and the posterior body (arrowheads) of the corpus callosum on diffusion-weighted images (D-F). The same regions are also shown in fused images combining FLAIR, T1WI, and DWI sequences. DWI: Diffusion-weighted image; FLAIR: Fluid-attenuated inversion recovery; WI: Weighted image.

Postoperative MRI demonstrated near-total resection of the target area, with infarction confined to the residual corpus callosum (Figure 3).

However, following the second resection, the patient newly presented with a disorder called abulia or akinetic mutism. The symptoms were as follows: the patient remained responsive to commands with nodding but could not speak (mutism); with persistent prompting, he retained the ability to transfer independently, ambulate with a walker, and perform oral hygiene, but he exhibited akinesia with minimal spontaneous movement. He also demonstrated instinctive grasping behavior, characterized by visual tracking of objects with subsequent compulsive grasping (visual probing) (Video 1). The patient did not have sufficient ability to perform standardized rehabilitation assessment batteries, except for simple tests using playing cards. Following the rehabilitation therapist's instructions, the patient was able to perform actions such as stacking and organizing playing cards, sorting them by color, and stacking cards with the same numbers. The abulic state persisted without improvement, resulting in permanent sequelae that necessitated transfer to a long-term care facility, with a Karnofsky performance status of 50. 

**Video 1:** Visual probing. The patient demonstrates instinctive grasping behavior toward objects placed within his visual field, persistently maintaining grip once acquired. Source: Video provided by Sugii N (corresponding author).

## Discussion

In glioma surgery, surgeons should aim for 'maximal safe resection' to optimize therapeutic efficacy while preserving neurological function [2,3]. Neurosurgeons generally follow two principles: (1) avoiding bilateral resection of homologous areas to prevent severe functional deficits, and (2) assuming that resection limited to contrast-enhancing lesions in glioblastoma will not cause new impairments. However, these principles are based on empirical rules, and no literature clearly supports them. In our case, although the contrast-enhanced lesion extended to both sides of the ACG, we believed complete resection of the contrast-enhanced area could be achieved without damaging the non-contrast-enhanced regions via the surgical corridor established during the first surgery. The development of postoperative abulia following the second surgery demonstrated the invalidity of the second principle in this instance.

Typical glioblastomas exhibit ring-enhancing lesions on MRI [8], with the center of the ring representing the necrotic core, which is presumed to be functionally inactive. In contrast, the contrast-enhanced area corresponds to microvascular proliferation and accurately reflects the characteristic histopathological features of glioblastoma [9]. While the contrast-enhanced region is generally considered nonfunctional, whether all neural functions are completely lost remains uncertain. For example, in studies using tractography, white matter fibers have been visualized within contrast-enhancing lesions in glioblastoma [10,11]. In this case, we were able to visualize part of the commissural fibers within the contrast-enhancing lesion in the corpus callosum (Figures 2F-2H). These findings indicate that at least part of the white matter fiber structure remains within the contrast-enhancing lesion. Moreover, recent studies have reported that preserved task-specific neural activity may be observed in glioblastoma-affected cortex, suggesting that baseline functions may persist even within contrast-enhanced lesions [12,13]. In our case, tumors in the right corpus callosum and bilateral ACG demonstrated relatively faint contrast enhancement compared to the strongly ring-enhancing lesion in the left corpus callosum. The possibility of residual neural function in these weakly enhancing areas cannot be excluded.

The lesions in this case were large, extending not only to both sides of the ACG but also to the medial frontal cortex and the dorsal anterior corpus callosum. It is important to note that the resulting disability may have been caused by a combination of factors related to the removal of these regions. However, we focused on the dACC. According to the triple-network model of intrinsic connectivity networks, three major large-scale brain networks are critical for cognition: the central executive network (CEN), default mode network (DMN), and salience network (SN) [6,14]. The CEN is activated to process goal-directed tasks or thoughts, whereas the DMN is engaged during internally focused, self-referential cognition; these two networks exhibit anti-correlated activity. The SN identifies the salience of external or internal stimuli and mediates transitions between the CEN and DMN. The core hubs of the SN include the dACC and anterior insula; disruption of these regions leads to SN dysfunction, an inability to switch to the CEN (i.e., persistent DMN dominance), and DMN hyperactivity, resulting in abulia, as seen in our case [5,6,15-18].

The severity of impairment caused by bilateral ACG damage varies depending on the context. For example, akinetic mutism occurs in patients with stroke affecting bilateral ACG lesions, but the symptoms are typically transient [19,20]. Bilateral anterior cingulotomy is occasionally performed for chronic intractable pain; however, this procedure generally results in only mild executive and attentional deficits [21-23]. We speculate that the difference in abulia severity between our case (severe and permanent) and other cases (mild and transient) depends on whether bilateral dACC function was completely abolished (totally resected) or partially preserved (incompletely damaged with preserved cortices). If comparable cases are encountered, resecting only the more extensively tumor-involved side followed by chemoradiation therapy may help preserve activities of daily living and cognitive function to the greatest extent possible.

## Conclusions

We encountered a rare case of glioblastoma associated with abulia following aggressive resection of bilateral ACG lesions. Although the resection targeted only contrast-enhancing tissue, involvement of the bilateral dACC, a key node of the salience network, likely contributed to severe cognitive and motivational dysfunction. Greater attention to functional neuroanatomy may help improve outcomes in complex glioblastoma cases, even when operating within contrast-enhancing tumor regions.
